# Environmental Exposures and Parkinson’s Disease

**DOI:** 10.3390/ijerph13090881

**Published:** 2016-09-03

**Authors:** Sirisha Nandipati, Irene Litvan

**Affiliations:** Department of Neurosciences Movement Disorders Center, University of California, San Diego, CA 92093, USA; sirisha.nandipati@kp.org

**Keywords:** environment and Parkinson’s disease, pesticides and Parkinson’s disease, toxins and Parkinson’s disease

## Abstract

Parkinson’s disease (PD) affects millions around the world. The Braak hypothesis proposes that in PD a pathologic agent may penetrate the nervous system via the olfactory bulb, gut, or both and spreads throughout the nervous system. The agent is unknown, but several environmental exposures have been associated with PD. Here, we summarize and examine the evidence for such environmental exposures. We completed a comprehensive review of human epidemiologic studies of pesticides, selected industrial compounds, and metals and their association with PD in PubMed and Google Scholar until April 2016. Most studies show that rotenone and paraquat are linked to increased PD risk and PD-like neuropathology. Organochlorines have also been linked to PD in human and laboratory studies. Organophosphates and pyrethroids have limited but suggestive human and animal data linked to PD. Iron has been found to be elevated in PD brain tissue but the pathophysiological link is unclear. PD due to manganese has not been demonstrated, though a parkinsonian syndrome associated with manganese is well-documented. Overall, the evidence linking paraquat, rotenone, and organochlorines with PD appears strong; however, organophosphates, pyrethroids, and polychlorinated biphenyls require further study. The studies related to metals do not support an association with PD.

## 1. Introduction

Parkinson’s disease (PD) affects millions of people around the world and is a multi-organ neurodegenerative process, affecting the nervous system, olfactory, and gastrointestinal tract. Patients’ motor features are characterized by bradykinesia with rigidity, resting tremor, or both caused by a lack of the neurotransmitter dopamine from death of dopaminergic cells in the substantia nigra. Involvement of non-dopaminergic neurons explains the significant non-motor features patients with PD experience including depression, cognitive decline, sleep, and autonomic dysfunction due to degeneration of serotonin, noradrenergic, and cholinergic neurons [1]. The etiology of PD has been difficult to elucidate and appears to be a complex interplay of genetic and environmental factors [2]. Genetic factors are discussed elsewhere [3,4].

PD is the result of several levels of cellular dysfunction, including mitochondrial [5], lysososomal and protease dysfunction [6,7], disorders of calcium homestasis [8], neuroinflammation [9], alpha synuclein aggregation [10], and oxidative stress [11]. It is theorized that there is a “dual-hit” caused by genetic predisposition and subsequent environmental factors interacting to create the cellular dysfunction that causes PD. Furthermore, Braak and colleagues hypothesize that pathologic agents penetrate the nervous system via the olfactory bulb, gut, or both and gradually spread to the nervous system causing the non-motor and motor features of PD [12]. While the pathologic agent has yet to be identified, several environmental exposures have been associated with PD.

In this review, we summarize and examine human epidemiologic and case-control evidence for individual environmental agents associated with PD. Primary human research articles from peer-reviewed scientific journals were found from databases including PubMed and Google Scholar by typing in search phrases such as “environment and Parkinson’s disease” and “case-control Parkinson’s” with each of the environmental agents until April 2016 (Table 1). All case-control human studies that examined specific agents (not groups of agents) with a sample size of cases greater than fifty were included (see Table 1). We did not perform a comprehensive review of laboratory data and instead selected relevant laboratory studies to enrich our discussion.

## 2. 1-Methyl-4-phenyl-1,2,3,6-tetrahydropyridine (MPTP)

We begin our discussion with MPTP, which is not a common environmental exposure but was the first recognized agent that led to an animal model of Parkinsonism. MPTP was associated with rapid-onset Parkinsonism when young drug abusers presented in Northern California with severe and irreversible Parkinsonism responsive to dopamine therapy [13]. Subsequently, MPTP was identified in synthetic heroin as the causative agent. MPTP crosses the blood brain barrier, and, in a two-step reaction, converts to MPP+, which has a high affinity for dopamine transporters, explaining its selective damage of dopaminergic neurons [14,15,16]. MPP damages neurons by inhibiting Complex 1 of the electron transport chain [17]. Animal studies, including non-human primates and rodents, showed MPTP produces striatal dopamine depletion with selective destruction of dopaminergic neurons in the substantia nigra. Unlike in idiopathic PD however, other areas of the brain are remarkably spared in MPTP-induced Parkinsonism including the locus ceruleus and other non-dopaminergic regions. In addition, follow up of the drug addicts exposed to MPTP who developed Parkinsonism revealed the stable, and not progressive, course of their symptoms, along with no Lewy Body pathology in postmortem studies [18]. Interestingly, postmortem tissue analysis has also revealed active, ongoing inflammation with the presence of microglia, extracellular melanin, and neurodegeneration years after MPTP exposure, leading to the theory of “long-latency neurotoxicity” after agent exposure [18]. Oxidative stress and ongoing inflammation leading to nigral cell loss have been hypothesized as the mechanism for the progressive and continued degeneration.

The study of MPTP has led to the development of the first animal models of the disorder and continued interest in compounds that protect against neuroinflammation as potential treatments of PD [19].

## 3. Pesticides

A frequent finding of epidemiologic studies is that PD is associated with farming as an occupation, rural living, and well-water exposure. Subsequent research has found that pesticides indeed can cause PD pathology in both animal models and humans.

Pesticides include an array of compounds designed to kill insects (insecticides), plants (herbicides), and fungi (fungicides). Over five billion pounds of pesticides were used worldwide in 2007 [20]. Human exposure occurs through ingestion of pesticide residues in food, drinking water, and most significantly, in occupational use including in agricultural field workers and workers in the pesticide industry. Assessment of occupational human exposure and risk is extremely difficult given the variability in protective measures during pesticide application, the dosage used, and the combination of pesticides used. Exposure in non-occupational settings has been even more difficult to determine. However, even with these limitations, many studies have found an association of PD with pesticides.

The first well-known study was in 1986, when Barbeau and colleagues evaluated PD prevalence in a homogenous population in Quebec, Canada, and found high rates of prevalence in areas of pesticide use [21]. This was followed by two case-control studies looking more closely at pesticide use, based on subject interview, with no significant association found between pesticide use and PD [22,23]. Another group chose to focus on young onset PD patients, as they hypothesized that those who presented with PD early in life may have been exposed to a higher dose of the causative environmental agent. They found that PD was associated with insecticide or herbicide exposure, past residency in a fumigated house, and rural residency at time of diagnosis [24]. The strengths of this study included validating the questionnaire they used for patient self-exposure before using it on all subjects, as well as examining the possibility of exposure in multiple forms of questions including asking about home fumigation. Another interesting study used local agricultural office pesticide records to tailor a structured interview for subjects, with cue cards showing the trade and common pesticide names, to more accurately determine whether subjects were exposed to specific pesticides [25]. Despite the relative wealth of specific pesticide data in this study, the overall use of pesticides was associated with PD, but the use of individual pesticides was not. Three other studies in the 1990s relying on subject self-reported pesticide use found no association between PD and pesticides [26,27,28], with another study finding a marginal association [29]. In 2001, a study more carefully assessed the years of exposure to pesticides and its association with Parkinsonism (as diagnostic data for PD was not sufficiently available) and found higher prevalence of PD in the highest tertile of years of pesticide exposure, but not in lesser durations, suggesting a dose-dependent relationship [30].

A seminal study examining duration of pesticide exposure was completed using data from the Agricultural Health Study (AHS), a large American cohort study of over fifty thousand pesticide applicators and their spouses, and found that PD was associated with higher cumulative days of pesticide exposure at study enrollment [31]. This study also evaluated 26 individual pesticides and their associations with PD, though most of these compounds were used by relatively few subjects (less than fifty), finding only four compounds significantly associated with PD. The major strength of this study is the large sample size, use of a cohort of pesticide applicators, and detailed associated data, but a significant weakness is the reliance on subject self-report for both pesticide data and history of PD. Another large United States cohort study found self-reported pesticide exposure to be significantly associated with PD, while other occupational exposures including solvents were not [32].

Finally, a recent study that avoided the use of subject self-report and used geographic pesticide exposure estimates (a similar approach to that used by other investigators, discussed further below [33,34,35,36]) and Nebraska’s state-wide PD registry to find a significant association between PD and certain pesticide ingredients that have not been previously well studied [37]. It is hoped that such novel approaches, compared to the standard use of self-report, will be continued in future studies. Another recent cohort study in the Netherlands found few weak associations between PD and exposure to pesticides, but no association with exposure duration or cumulative exposure [38].

A limitation of these studies is that they often examine entire classes of pesticides and have limited data on specific agents. Though the cumulative and perhaps synergistic effects of pesticides should be explored, here we focus our review on a smaller number of studies that examine individual pesticide compounds and their association with PD.

### 3.1. Rotenone

Rotenone is a plant-derived, naturally occurring pesticide that is commonly used as an insecticide and to kill fish in reservoirs. Though it is organic and thus originally thought to be non-toxic to humans, it has been found to be a mitochondrial toxin that inhibits Complex I of the electron transport chain [39] with resulting progressive neurodegeneration of dopaminergic and non-dopaminergic neurons and oxidative damage [40,41,42]. In rat models, administration of rotenone by daily intraperitoneal injection produced bradykinesia, postural instability, and rigidity responsive to dopamine. Postmortem rat studies found that nearly half of substantia nigra and striatal neurons were lost. In addition, alpha-synuclein and polyubiquitin positive aggregates were observed in dopamine neurons in the substantia nigra, similar to the Lewy Bodies found in PD [40,43] and in the enteric nervous system [44,45]. Rotenone can also lead to phosphorylation and aggregation of tau and amyloid proteins [46,47,48].

However, it is not always clear if the motor decline associated with rotenone is due solely to dopaminergic dysfunction. Other animal studies of rotenone have found that, after low to high doses of rotenone administration, motor decline is not always associated with dopaminergic cell loss, suggesting that rotenone may cause diffuse mitochondrial dysfunction in central non-dopaminergic as well as peripheral cells outside of the central nervous system [49,50].

Now that rotenone-exposed animal models are reproducible and respected as a laboratory model of PD, recent rotenone research focus is on new treatment. For example, a dietary phytocanniboid given to rats before exposure to rotenone has been found to reduce oxidative damage, glial activation, and dopaminergic cell loss [51]. Another study found that an adenosine receptor antagonist increased midbrain dopamine concentrations and reduced the motor slowing in rotenone-exposed rats [52].

Human epidemiological studies show that PD developed more often in people who reported use of rotenone compared with nonusers [53,54]. Among the most well known studies are those performed in the large AHS cohort [53]. In addition, the Farming and Movement Evaluation study (FAME), a small nested study within the AHS with only 69 cases and 237 controls, determined that rotenone was associated with PD regardless of the use of protective gloves [55]. Despite the mixed laboratory results, the supportive human epidemiologic studies combined with many supportive laboratory studies suggest that rotenone is likely associated with PD.

### 3.2. Paraquat & Maneb

The herbicide paraquat is an oxidative stressor that, in several human case-control studies, was associated with PD [31,34,35,53,56]. There has been great interest in paraquat due to its chemical structure closely resembling the active metabolite of MPTP. Paraquat has often been studied with maneb, a fungicide often used in the same geographical regions, and the evidence suggests these pesticides likely have synergistic effects [57,58]. Maneb has been studied little individually, but, in one early study, has been associated with Parkinsonism in humans [59]. Paraquat causes tissue damage by setting off a redox cycle that generates toxic superoxide free radicals. A mimetic of superoxide dismutase, an enzyme key in neutralizing superoxide free radicals, has been found to reduce the mitochondrial damage and motor slowing cause by paraquat in *Drosophila* flies [60]. Furthermore, in vivo animal studies indicate that paraquat stimulates glutamate efflux initiating excitotoxicity mediated by reactive nitrogen species [61]. Similar studies have also demonstrated that paraquat induces alpha-synuclein upregulation [62], aggregate formation, and microglial activation [63,64]. Systemic subchronic exposure to paraquat in mice induces dopaminergic neuronal cell death in the basal ganglia, though overall dopamine levels remained unchanged [65]. However, higher doses of chronic exposure did cause slow progressive degeneration of nigrostriatal neurons and delayed reduction of dopaminergic neurotransmission [66], suggesting that paraquat may cause a “subclinical” insult, and additional environmental or genetic factors may be required for PD to develop. In fact, in another AHS epidemiologic study, deletions in the gene for a glutathione transferase, an enzyme that provides cellular protection against oxidative stress, was associated with a higher risk of PD when male subjects reported exposure to paraquat [67]. This area requires further investigation in humans since this study was limited by a small sample size and exposure to multiple pesticides in addition to paraquat. One more human study found that dopamine transporter susceptibility alleles and exposure to maneb and paraquat led to higher PD risk [36], another example of the dual-hit phenomenon and possible synergism. Strengths of this study included the use of geographic exposure estimates instead of subject self-report, but the study was also limited by smaller subject sample sizes.

An alternative theory is that prenatal or early exposure to paraquat and maneb may disrupt the development of the nigrostriatal dopamine system and enhance susceptibility to neurotoxicant exposures later in life [68,69]. In support of this theory, the offspring of pregnant mice treated with maneb had normal locomotor activity, but after subsequent treatment with paraquat, they had significant reductions in locomotor activity and selective dopaminergic loss in the substantia nigra. However, mice treated with paraquat prenatally and then maneb later in life did not show the same changes, suggesting that the specificity and sequence of exposure may be important factors. Other studies have found synergy between paraquat and other compounds including MPTP [70] and the organophosphate insecticide chlorpyrifos [71].

Beyond animal studies, human epidemiological studies have found that not only is paraquat associated with PD occurrence, but the incidence of disease and the extent of paraquat exposure can sometimes strongly correlate [56]. However, the human evidence has been mixed. In addition to the negative pesticide studies described previously, a French case-control human study looking at specific pesticide exposures found that paraquat was not associated with PD, but this was possibly due to the study being performed in France, where paraquat is used at lower levels [72]. Like rotenone, all research has not been entirely consistent, but overall several human case-control studies and laboratory studies support an association between paraquat, with evidence of synergism with other compounds such as maneb, and PD.

### 3.3. Organochlorines

Organochlorine pesticides are chlorinated hydrocarbons used extensively in the 1940s through the 1970s in agriculture and mosquito control, and they have been banned in the United States since they were suspected to be neurotoxicants [73] and have been associated with PD [72,74]. Two compounds in particular, dieldrin and β Hexachlorocyclohexane (HCH), have been implicated as associated with PD [75,76,77,78]. Both are lipophilic compounds that can be easily absorbed through the skin, stored in fatty tissues for extended periods of time, and penetrate the blood brain barrier. Dieldrin is thought to contribute to cell death in the substantia nigra by impairing mitochondrial function and creating oxidative stress via reactive oxygen species [79,80] when the exposure is to relatively high concentrations, but these effects are weak compared with those of rotenone, which requires relatively low concentrations to cause such effects. However, an in vitro study did find that dieldrin and HCH disrupt calcium homeostasis of dopaminergic cells even at low, nanomolar concentrations [81], suggesting that further in vivo study should be performed. A case-control study that analyzed serum samples for five organochlorine pesticides found only dieldrin to be associated with a higher risk of PD [82]. In another case-control study, a genetic polymorphism associated with a decreased ability to clear toxins from the brain and professional organochlorine exposure were associated with increased PD risk [83]. Of note, postmortem studies of human brains have also found a higher concentration of organochlorine compounds in PD brains, specifically in the striatum, compared with the brains of patients without PD [84,85], while another postmortem study found organochlorine levels were nonsignificantly associated with Lewy bodies [86].

### 3.4. Organophosphates

Organophosphates are pesticides that have known acute neurotoxic effects. However, chronic professional and even household exposure may lead to increased PD risk [87,88]. In animals, neonatal exposure to the chlorpyrifos led to long-term dopaminergic cell loss and microglial activation [89]. A case-control study found that higher rates of ambient organophosphate exposure were associated with higher PD risk [33]. Interestingly, a common genetic variant of an enzyme important in detoxifying organophosphates was also found to be associated with a greater than two-fold increased risk in PD when carriers had been exposed to organophosphates compared with subjects without the variant genotype [90]. The genetic variant has also been associated with PD in genetic analysis comparing PD subjects to normal controls [91,92]. Many organophosphates, when entering the bloodstream, are bioactivated into a toxic oxon form. Any oxon that escapes liver detoxification can be hydrolyzed in the blood by serum paraoxonase (PON1) before it reaches the brain. The genetic variability between individuals in PON1 activity may therefore lead to variable vulnerability to organophosphate neurotoxic effects [90,92].

### 3.5. Pyrethroids

Pyrethroids are a newer class of insectides often contained in household insecticides and mosquito repellants. Animal studies have reported the ability of pyrethroids to indirectly increase dopamine transporter-mediated dopamine uptake [93] and thus cause indirect apoptosis of dopaminergic cells [94,95]. However, this area requires further study, as there is little specific human data besides the general finding that pesticide exposure, including pyrethroids, is associated with PD [73].

## 4. Metals

Humans are exposed to metals through a variety of sources, including through diet and occupational exposures such as manufacturing and welding. Many metals are essential minerals important for human health. However, too much metal can be detrimental, and we have chosen to focus on two metals in particular, iron and manganese, that have been associated with PD.

### 4.1. Iron

Iron has been found to be likely linked to the pathophysiology of PD in several laboratory studies, but information of human exposure is scarce [4,96]. Some studies have found that increased dietary iron is associated with PD [97,98], while studies on serum iron levels and their association with PD have been conflicting [99,100,101,102]. Substantia nigra neurons contain neuromelanin that can bind to iron and produce free radicals that in turn initiate lipid peroxidation and cell death [103]. Iron also promotes auto-oxidation of dopamine in substantia nigra neurons, releasing additional free radicals [104]. Pathological studies have found increased levels of iron in the substantia nigra of PD patients [105], and animal studies have shown that, in MPTP-induced Parkinsonism, iron chelation can be therapeutic [106]. However, it remains unclear whether iron accumulation precedes injury of substantia nigra neurons or occurs as a consequence of neuronal degeneration [103].

### 4.2. Manganese

Manganese is a basal ganglia toxin associated with Parkinsonism, but its association to idiopathic PD remains controversial [107,108]. Manganese exposure can occur in working with steel, battery manufacturing, intravenous synthetic drug use, and long-term parental nutrition and causes a distinct syndrome with postural instability, a high-stepping gait characterized as a “cock-walk”, bradykinesia, and rigidity that does not consistently respond to L-dopa therapy [108,109,110,111,112]. Such manganese-induced Parkinsonism is distinct from idiopathic PD, which characteristically has bradykinesia and rigidity that responds well to L-dopa with postural stability not presenting until later stages of the disease. In manganese-induced Parkinsonism, MRI reveals T1 hyperintensity in the striatum and globus pallidus and a normal dopamine transporter (DaT) scan, unlike in idiopathic PD when there are no MRI changes and an abnormal DaT scan.

The mechanism of manganese neurotoxicity is degeneration of the globus pallidum mediated by disruption of the mitochondria initiating both apoptosis and cell death via formation of highly reactive oxygen species [113]. Manganese is rapidly taken up by the mitochondria where it promotes calcium accumulation and inhibits oxidative phosphorylation, resulting in depletion of ATP [114,115,116]. In addition, manganese inhibits glutamate transport leading to increased levels of glutamate and thus cytotoxicity [117]. In rats, early low-level exposure to manganese was associated with higher levels of astrocytosis in the striatum as well as motor and cognitive impairment later in life, supporting manganese as a potent neurotoxicant [118].

Despite the distinction between manganese neurotoxicity and idiopathic PD, there continues to be interest in manganese exposure being a risk factor for PD with a few supportive studies. A population-based case-control study found that a small percentage of subjects who reported over twenty years of occupational exposure to manganese had a significantly higher risk of PD [119]. PD incidence was also found to be higher in urban counties with documented increased manganese industrial emissions [120] and in subjects who consumed higher amounts of dietary iron and manganese [121]. An area of significant controversy is the association between PD and welding, with reports suggesting that welding is associated with significant manganese exposure and may cause Parkinsonism [122,123,124,125], with some investigators claiming that this Parkinsonism overlaps with PD with no proof of true PD in these studies. Overall review of the evidence suggests that there is no clear association between welding and PD [126], but manganese-induced Parkinsonism in welding may be a true phenomenon difficult to disentangle from its impact in sporadic PD in view of litigations related to job-related manganese exposure and PD.

## 5. Polychlorinated Biphenyls (PCBs)

Polychlorinated Biphenyls (PCBs) were produced and used commercially in industrial manufacturing in the 1930s–1970s. They are lipophilic compounds that have been found in the fatty tissues of fish and marine mammals [127]. In monkeys, PCB exposure produced a decrease of dopamine in the brain [128]. The mechanism is thought to be the downregulation of dopamine transporters that precedes damage to the dopamine striatal system [129]. In multiple human studies, increased amounts of PCBs have been found in postmortem studies of PD brain tissue compared with brain tissue unaffected by PD [84,130]. Humans who consumed higher amounts of whale were also found to be at risk for PD [131]. However, other studies examining PCB exposure history and serum levels of PCBs before subjects develop PD have found no association with PD [132,133]. Overall, the human data is mixed. Though the commercial use of PCBs has been eliminated, their persistence in the environment and the human body, with primary exposure through diet especially from the consumption of marine mammals, make them a lasting threat to vulnerable populations such as the Inuit.

## 6. Gaps in Knowledge

Several questions remain and require further study. While there has been significant work in how environmental substances disrupt dopaminergic neurotransmission, more study is needed on how these substances affect non-motor PD symptoms and disrupt related neurotransmitters including noradrenaline, serotonin, and acetylcholine. Similarly, it would be critical to determine if there is an association between prodromal PD [134] and exposure to currently used pesticides. More studies are needed of the synergistic effects of multiple compounds as well as the interaction of pesticide exposure with PD genetic predisposition. This will require large, multicenter, sufficiently powered studies that use the same methodology. In addition, there is inadequate understanding on how toxins can affect the microbiota or olfactory bulb and lead to alpha-synuclein aggregation, although we discussed evidence of pesticides causing peripheral motor deficits and lung damage. We also need more objective quantification of patient exposure, using geographic estimates, laboratory assays, and better tracking by government agencies of pesticide and industrial compound use, because too much of our current work relies on subject self-report, which is inevitably susceptible to bias and human error. Finally, positive laboratory studies should be treated with measured skepticism, as varying techniques (in vitro versus in vivo) and concentrations of toxins can produce contradictory and misleading results.

Clinicians, patients, and occupational health professionals need to better understand how to apply the current knowledge to making clinical recommendations and lifestyle changes. We need to understand how important a single environmental exposure is in relation to one’s genetic predisposition and the infinite exposures one has in a lifetime, and how these factors can be modified to prevent the development of PD. We need to understand how much exposure of a certain agent is hazardous because most of the data we have is limited to robust and substantial exposures and not lower rates of exposure, and whether the effects of toxicants can be synergistic. Ultimately, the most intriguing question is whether laboratory studies of these compounds can lead to an accurate disease model that could lead to the discovery of disease-modifying therapies. Environmental exposures and PD is an exciting field for further study.

## 7. Conclusions

Based on our detailed review of several original research studies, the evidence is strong that environmental exposures play a role in the etiology of PD. Specifically, rotenone, paraquat, and organochlorines have been well-documented in human epidemiological studies to be associated with PD. Adding to the evidence, rotenone and paraquat often produce both the symptomatology and pathology of PD in the laboratory, though importantly there are negative laboratory studies and studies that show that rotenone and paraquat can cause peripheral injury that causes locomotor deficits, as opposed to only central nervous system disease. In case-control studies, it is remarkable how substantial exposure for long durations, related to farming as an occupation, suggests an association with PD, but we have fewer data on lower rates of exposure. Organophosphates, pyrethroids, and PCBs require further study since human data are limited. The studies related to metals have overall been inconclusive or do not support an association with PD, and review of this literature highlights the importance of separating PD and substance-induced Parkinsonism in evaluating environmental exposures.

## Figures and Tables

**Table 1:** Summary of Case-Control Human Studies. Please see separate revised file in online submission.

Environmental Agent	Authors	Number of Cases/Controls	Method	Conclusions
Pesticides	Barbeau et al. 1987 [21]	5270 cases	Data analysis of geographic incidence of PD, pesticide sales and mapping of hydrographic regions	Pesticide use significantly correlated with PD prevalence (*r* = 0.967)
	Stern et al. 1991 [23]	161/149	Chart review and Interview	Exposure to pesticides were not associated with PD
	Jimenez-Jimenez et al. 1992 [22]	128/256	Questionnaire and neurologic assessment	PD not associated with history of pesticide exposure
	Butterfield et al. 1993 [24]	63/68	Questionnaire and Interview	Herbicide (OR 3.22) ** and insecticide (OR 5.75) *** exposure each associated with risk of PD
	Hertzman et al. 1994 [25]	127/245	Interview and neurologic assessment	Occupational exposure to pesticides significantly associated with risk of PD in male subjects OR 2.03 (95% CI 1.0, 4.12), with no significant association found with specific pesticides
	Morano et al. 1994 [28]	74/148	Questionnaire	PD not associated with history of pesticide exposure, though well water drinking and rural living was.
	Chan et al. 1998 [29]	215/313	Questionnaire, neurologic assessment and genetic testing	Duration of pesticide exposure associated with marginally increased risk of PD OR 1.05 (95% CI 1.01, 1.09) *
	McCann et al. 1998 [26]	224/310	Interview and neurologic assessment	PD not associated with history of pesticide exposure
	Kuopio et al. 1999 [27]	123/145	Interview and neurologic assessment	No significant association between pesticides and PD.
	Engel et al. 2001 [30]		Questionnaire and neurologic assessment	PR of 2.0 (95% CI 1.0, 4.2) ** for subjects in the highest tertile of years of exposure to pesticides and a similarly increased, non-significant PR was found for the middle tertile. No increased risks were found associated with specific pesticides.
	Ascherio et al. 2006 [32]	7864	Questionnaire and medical record review	Exposure to pesticides had a 70% higher incidence of PD than in those without exposure **
	Kamel et al. 2007 [31]	161/55,931	Questionnaire and Interview	PD associated with cumulative days of pesticide use at enrollment, OR 2.3 (95% CI 1.2, 4.5) **
	Brouwer et al. 2015 [38]	609/4391	Questionnaire and cohort follow up of PD incidence	Few significant associations between PD and occupational exposure to pesticides
	Wan et al. 2015 [37]	6557 cases	Use of state-wide PD registry and geographic estimates of pesticide exposure	No significant association with paraquat exposure, but with other less studied pesticide ingredients
Rotenone	Tanner et al. 2011 [51]	110/358	Interview and neurologic assessment	Rotenone exposure associated with PD, OR 2.5 (95% CI 1.3, 4.7) **
	Dhillon et al. 2008 [52]	102/84	Questionnaire	Report of past rotenone use was associated with PD, OR 10.0 (95% CI 2.9, 34.3)
	Furlong et al. 2015 [53]	69/237	Nested Case Control Study	Protective glove use modified association of paraquat and permethrin with PD, paraquat OR 3.9 (95% CI 1.3, 11.7) * & permethrin OR 4.3 (95% CI 1.2, 15.6) * but did not modify the association with rotenone
Paraquat	Liou et al. 1997 [54]	120/240	Interview and neurologic assessment	Paraquat exposure associated with PD, OR 3.2 (95% CI 2.41, 4.31) **
	Kamel et al. 2007 [31]	14/11,266	Questionnaire and Interview	Paraquat associated with higher rate of prevalent PD, O.R. 1.8 (95% CI 1.0, 3.4)
	Costello et al. 2009 [34]	268/341	Interview and geographic estimates of ambient paraquat exposure	Paraquat exposure associated with increased PD risk, OR 2.27 (95% CI 0.91, 5.70)
	Ritz et al. 2009 [36]	324/334	Genetic testing, neurologic assessment and geographic exposure estimates	PD risk was increased in subjects who had one ore more dopamine transporter susceptibility alleles with high exposure to paraquat and maneb 1 allele OR 2.99; (95%CI 0.88, 10.2) & >2 alleles OR 4.53 (95% CI 1.7, 12.1)
	Elbaz et al. 2009 [68]	224/557	Clinical evaluation and interview	Paraquat exposure not associated with PD
	Tanner et al. 2011 [51]	110/358	Interview and neurologic assessment	Paraquat exposure associated with PD, OR 2.0 (95% CI 1.4, 4.7) **
	Lee et al. 2012 [35]	357/754	Clinical evaluation, interview and pesticide exposure estimate	Paraquat exposure and history of traumatic brain injury associated with PD risk OR 2.77 (95% CI 1.45, 5.29)
	Goldman et al. 2012 [64]	87/343	Interview, neurologic assessment and DNA analysis	Men exposed to paraquat with functional glutathione S-transferase M1 (GSTT1) genotype had lower risk of PD compared to men exposed to paraquat lacking GSTT1
Maneb	Ferraz et al. 1988 [57]	50/19	Questionnaire and neurologic assessment	Increased cogwheel rigidity associated with maneb exposure **
	Wang et al. 2011 [56]	362/341	Interview and geographic estimates of ambient paraquat, maneb and ziram exposure	Combined exposure to all 3 pesticides associated with PD risk at workplaces OR 3.09 (95% CI 1.69, 5.64) and residences OR 1.86 (95% CI 1.09, 3.18) and combined exposure to ziram and paraquat at workplaces associated with PD risk OR1.82 (95% CI 1.03,3.21)
Organochlorines	Seidler et al. 1996 [70]	380/755	Clinical evaluation and interview	Association between PD and organochlorine exposure OR 5.8 (95% CI 1.1, 30.4)
	Richardson et al. 2009 [71]	50/43	clinical evaluation and serum testing for levels of organlchlorine pesticides	β-HCH was associated with higher likelihood of PD, OR 4.39 (95% CI 1.67, 11.6) ***
	Elbaz et al. 2009 [68]	224/557	Clinical evaluation and interview	Organochlorine exposure associated with PD in men OR 2.2 (95% CI 1.1, 4.3)
	Dutheil et al. 2010 [78]	207/482	Clinical evaluation, questionnaire and DNA analysis	Homozygous variants in the ABCB1 gene, responsible for clearing xenobiotics, and reported organochlorine exposure was associated with PD O.R 3.5 (95% CI 0.9, 14.5)
	Weisskopf et al. 2010 [77]	349/101	Nested case-control study within Finnish Mobile Clinic Health Examination survey with analysis of serum samples for dieldren	Dieldren was associated with OR 1.28 (95% CI 1.26, 3.02) **
	Richardson et al. 2011 [71]	149/134	Clinical evaluation and serum testing for levels of organochlorine pesticides	PD patients had higher serum levels of β-HCH than controls in higher exposure cohort **, but in cohort with lower levels there was no significant difference
	Webster Ross et al. 2012 [81]	225	Postmortem study of organochlorine levels in frozen occipital lobe samples and identification of Lewy Bodies and Lewy neurits	Insignificant associations between Lewy Body pathology and presence of organochlorine compounds
	Chhillar et al. 2013 [73]	70/75	Clinical evaluation and serum testing for levels of organochlorine pesticides	β Hexachlorocyclohexane (HCH) and Dieldren levels were significantly higher in PD *** with OR 2.56 (95% CI 1.68, 3.91) & 2.09 (95% CI 1.41, 3.11)
	Steenland et al. 2014 [74]	89	Clinical evaluation and serum testing for levels of organochlorine pesticides	Dieldrin was associated with a nonsignificant higher risk of tremor at rest
Organophosphates	Akhmedova et al. 2001 [86]	117/207	DNA sample analysis	Association between PD and *PON1* gene polymorphism **
	Carmine et al. 2002 [85]	114/127	DNA sample analysis	Association between PD and *PON1* gene polymorphism *
	Firestone et al. 2005 [82]	250/388	Interview and chart review	Organophosphate parathion associated with PD OR 8.08 (95% CI 0.92, 70.85)
	Manthripragada et al. 2010 [84]	351/363	Interview, estimate of ambient pesticide exposure and DNA sample analysis	Increased risk of PD with exposure to ambient organophosphates and having common genetic variant in *PON1*
	Wang et al. 2014 [33]	357/752	Interview and geographic estimates of ambient pesticide exposure	Exposure to ambient organophosphates associated with increased odds of PD
	Narayan et al. 2013 [83]	357/807	Interview and home pesticide ingredient database review	Frequent use of household pesticides containing organophosphates increased the odds of PD more strongly by 71% OR 1.71 (95% CI 1.21, 2.41)
Iron	Logroscino et al. 2008 [91]	422/124,353	Questionnaire	Dietary nonheme iron intake associated with PD, relative risk 1.27 (95% Cl 0.92, 1.76) *
	Miyake et al. 2011 [90]	249/368	Clinical evaluation and questionnaire	Higher dietary intake of iron and other metals associated with lower risk of PD OR 0.33 (95% CI 0.13, 0.81) **
	Farhoudi et al. 2012 [94]	50/50	Serum sample analysis	Serum iron levels were not significantly different between PD and control subjects
	Zhao et al. 2013 [93]	238/302	Clinical evaluation and blood sample analysis	Iron and selenium concentrations were significantly increased in PD patients **
	Kumudini et al. 2014 [92]	150/170	Clinical evaluation and blood sample analysis	Plasma iron and copper levels were significantly elevated * in PD subjects compared to controls, with no significant difference in manganese and lead
	Costa-Mallen et al. 2015 [95]	128/226	Serum iron, ferritin and haptoglobin phenotype testing	PD cases has lower serum iron levels than controls
Manganese	Gorell et al. 1997 [111]	144/464	Survey and estimates of occupational metal exposure	No significant elevated risk of PD with estimated manganese exposure
	Powers et al. 2003 [113]	250/388	Interview and nutrient intake estimates	High intake of iron with manganese associated with increased PD risk
	Park et al. 2004 [118]	105/129	Interview and questionnaire	Occupations with high potential exposure to manganese not significantly associated with PD
	Willis et al.. 2010 [112]	NA	PD incidence calculated and compared between counties with high or low industrial release of manganese	PD incidence was greatest in counties with high manganese release
Polychlorinated biphenyls (PCBs)	Steenland et al. 2005 [125]	N/A	Retrospective data analysis	No overall increased incidence of PD in PCB exposed workers
	Petersen et al. 2008 [123]	79/154	Clinical evaluation and serum and hair testing	Whale meat consumption significantly associated with PD, OR 6.53 (95% CI 3.02, 14.14) ** serum PCBs not associated with PD
	Weisskopf et al. 2012 [124]	101/349	Nested case-control study within Finnish Mobile Clinic Health Examination survey with analysis of serum samples for PCBs	No significant association between increasing PCB serum levels and PD

PD: Parkinson’s disease. OR = Odds ratio, CI = Confidence Interval, * *p* < 0.05, ** *p* < 0.01, *** *p* < 0.001, PCB = polychlorinated biphenyls.
